# Rpl22 is required for *IME1* mRNA translation and meiotic induction in *S. cerevisiae*

**DOI:** 10.1186/s13008-016-0024-3

**Published:** 2016-07-29

**Authors:** Stephen J. Kim, Randy Strich

**Affiliations:** Department of Molecular Biology, Rowan University School of Osteopathic Medicine, Two Medical Center Dr., Stratford, NJ 08055 USA

**Keywords:** Translation, Differentiation, Meiosis, Ribosome

## Abstract

**Background:**

The transition from mitotic cell division to meiotic development in *S. cerevisiae* requires induction of a transient transcription program that is initiated by Ime1-dependent destruction of the repressor Ume6. Although *IME1* mRNA is observed in vegetative cultures, Ime1 protein is not suggesting the presence of a regulatory system restricting translation to meiotic cells.

**Results:**

This study demonstrates that *IME1* mRNA translation requires Rpl22A and Rpl22B, eukaryotic-specific ribosomal protein paralogs of the 60S large subunit. In the absence of Rpl22 function, *IME1* mRNA synthesis is normal in cultures induced to enter meiosis. However, Ime1 protein production is reduced and the Ume6 repressor is not destroyed in *rpl22* mutant cells preventing early meiotic gene induction resulting in a pre-meiosis I arrest. This role for Rpl22 is not a general consequence of mutating non-essential large ribosomal proteins as strains lacking Rpl29 or Rpl39 execute meiosis with nearly wild-type efficiencies. Several results indicate that Rpl22 functions by enhancing *IME1* mRNA translation. First, the Ime1 protein synthesized in *rpl22* mutant cells demonstrates the same turnover rate as in wild-type cultures. In addition, *IME1* transcript is found in polysome fractions isolated from *rpl22* mutant cells indicating that mRNA nuclear export and ribosome association occurs. Finally, deleting the unusually long 5′UTR restores Ime1 levels and early meiotic gene transcription in *rpl22* mutants suggesting that Rpl22 enhances translation through this element. Polysome profiles revealed that under conditions of high translational output, Rpl22 maintains high free 60S subunit levels thus preventing halfmer formation, a translation species indicative of mRNAs bound by an unpaired 40S subunit. In addition to meiosis, Rpl22 is also required for invasive and pseudohyphal growth.

**Conclusions:**

These findings indicate that Rpl22A and Rpl22B are required to selectively translate *IME1* mRNA that is required for meiotic induction and subsequent gametogenesis. In addition, our results imply a more general role for Rpl22 in cell fate switches responding to environmental nitrogen signals.

## Background

The budding yeast *S. cerevisiae* chooses alternative cell fates based on cell type and environmental cues. For example, in response to poor nitrogen sources, haploid and diploid yeast will undergo a dimorphic switch leading to invasive or pseudohyphal growth, respectively. The switch to pseudohyphal growth requires Ras signaling through Protein Kinase A and is inhibited in response to available nitrogen by Tor1 kinase activation [1–4]. Similarly, meiotic induction occurs only in diploid cells deprived of nitrogen and a fermentable carbon source [5]. The switch from mitotic to meiotic cell divisions requires expression of *IME1*, which induces the meiotic transcription program by binding and triggering the destruction of the Ume6 repressor [6]. Interestingly, Ime1 is also required for pseudohyphal growth [7] suggesting that the regulatory pathways controlling these two processes exhibit some degree of overlap. *IME1* transcription is controlled by a complex and extensive set of cis-acting promoter elements that respond to cell type, carbon and nitrogen signals [8, 9].

In addition to transcriptional control, *IME1* mRNA translation is restricted to meiosis although specific mechanisms were not identified [10, 11]. Many translational control mechanisms in eukaryotic cells operate during translation initiation focusing on the formation of a stable pre-initiation complex. Once a stable complex is formed between the mRNA, the 40S subunit and the initiator tRNA, the catalytic 60S large subunit associates with the small subunit to form the functional 80S complex capable of translation [12, 13]. Following formation of a stable pre-initiation complex, translation can still be inhibited through other mechanisms. For example, the presence of short, upstream open reading frames (uORFs) before the protein encoding initiating AUG causes ribosome stalling and disassociation [14, 15].

The roles that ribosomal proteins (RPs) themselves play in regulating translation initiation are less well understood. The ribosome is composed of an rRNA core bound by many RPs that play essential structural roles for ribosome assembly and function [16–18]. Of the 78 ribosomal protein families in eukaryotes, 34 are also found in prokaryotic ribosomes, 67 in archaea [18] leaving only 11 families that are specific to eukaryotic cells [19]. Despite the critical role of translation for cellular function, 14 RPs in yeast are not essential for viability indicating that not all ribosomal proteins serve a basic translation function [18].

One of the non-essential RPs only found in eukarya is the large subunit protein family L22e. *RPL22* exist as a paralog pair in yeast (*RPL22A*, *RPL22B*) and mammals (Rpl22, Rpl22-like) [20, 21]. L22e binds a stem-loop on the rRNA [16, 22]. However, it is neither directly at the interface of the ribosomal subunits, nor does it play a structural role in organizing the protein exit channel [16, 17]. The murine Rpl22 is not essential for viability but is required for the differentiation of αβ T-cells in mice and hematopoietic stem cell emergence in zebrafish indicating it plays a more specialized role in cell fate decisions [23, 24]. Another group has shown differential expression of Rpl22 and Rpl22-like, the latter of which is alternatively spliced in Drosophila spermatocytes [25]. Interestingly, Rpl22 inhibits the expression of Rpl22-like1 in mice suggesting antagonistic functions for these proteins [20]. The current study describes a role of Rpl22 in mediating cell fate decisions in budding yeast. Although a modest defect is observed in mitotic cell division, loss of Rpl22 function results in significant defects in both pseudohyphal growth and execution of meiotic divisions. The latter phenotype is due to the requirement of Rpl22 in translating the mRNA of the *IME1* meiotic inducer. These results identify a specific translation role for Rpl22 during yeast cell fate decisions.

## Methods

### Strains and plasmids

Strain genotypes are listed in Table 1. Haploid *rpl22AΔ* and *rpl22BΔ* strains were generated by transforming in PCR amplified KanMX cassettes from the Research Genetics collection of nonessential gene deletions into haploid yeast through homologous recombination. Deletion of both paralogs was accomplished through mating, and subsequent tetrad dissection. Homozygous diploids were generated by introducing *HO* expressing plasmids. The triple hemagglutinin (3HA) tagged *IME1* allele was constructed by transforming in linearized pSK17, which contains the last 500 bp of the *IME1* ORF in frame with 3HA, and the native 3′UTR on pRS306 [26]. The insertion was counter-selected on 5-fluororotic acid (5-FOA) to loop out the plasmid backbone. Individual isolates that retained the epitope tag were confirmed by sequence analysis of genomic PCR fragments. Similarly, the *IME1* 5′UTR was chromosomally deleted by transformation with pSK18, which has the *IME1* locus from −1 kb to +500, but lacking 180 bp in the 5′UTR in pRS306. The final deletion was also generated by counter selection on 5-FOA and verified by genomic sequencing.

**Table 1:** Strains used in this study

Strain	Genotype^a^	Reference
RSY333	*MAT* **a** *cyh2* ^R^-*z* *ho*::*LYS2* *leu2*::hisG *lys2* *trp1*::hisG *ura3*	[6]
RSY335	*MAT* **a**/*MAT*α *cyh2* ^R^-*z* *ho*::*LYS2* *leu2*::hisG *lys2* *trp1*::hisG *ura3*	[27]
RSY1446	*MAT* **a** *cyh2* ^R^-*z* *ho*::*LYS2* *leu2*::hisG *lys2* *trp1*::hisG *ura3* *rpl22B*::KANMX	This study
RSY1479	*MAT* **a** *cyh2* ^R^-*z* *ho*::*LYS2* *leu2*::hisG *lys2* *trp1*::hisG *ura3* *rpl22A*::KANMX	This study
RSY1483	*MAT* **a** *cyh2* ^R^-*z* *ho*::*LYS2* *leu2*::hisG *lys2* *trp1*::hisG *ura3* *rpl22A*::KANMX *rpl22B*::KANMX	This study
RSY1559	*MAT* **a**/*MAT*α *cyh2* ^R^-*z* *ho*::*LYS2* *leu2*::hisG *lys2* *trp1*::hisG *ura3* *rpl22A*::KANMX	This study
RSY1560	*MAT* **a**/*MAT*α *cyh2* ^R^-*z* *ho*::*LYS2* *leu2*::hisG *lys2* *trp1*::hisG *ura3* *rpl22B*::KANMX	This study
RSY1561	*MAT* **a**/*MAT*α *cyh2* ^R^-*z* *ho*::*LYS2* *leu2*::hisG *lys2* *trp1*::hisG *ura3* *rpl22A*::KANMX *rpl22B*::KANMX	This study
RSY1833	*MAT* **a**/*MAT*α *cyh2* ^R^-*z* *ho*::*LYS2* *leu2*::hisG *lys2* *trp1*::hisG *ura3* *IME1*::3HA	This study
RSY1839	*MAT* **a**/*MAT*α *cyh2* ^R^-*z* *ho*::*LYS2* *leu2*::hisG *lys2* *trp1*::hisG *ura3* *rpl22A*::KANMX *rpl22B*::KANMX *IME1*::3HA	This study
RSY1991	*MAT* **a**/*MAT*α *cyh2* ^R^-*z* *ho*::*LYS2* *leu2*::hisG *lys2* *trp1*::hisG *ura3* 5′UTRΔ-*IME1*:3HA	This study
RSY1993	*MAT* **a**/*MAT*α *cyh2* ^R^-*z* *ho*::*LYS2* *leu2*::hisG *lys2* *trp1*::hisG *ura3* *rpl22A*::KANMX *rpl22B*::KANMX 5′UTRΔ-*IME1*::3HA	This study
RSY883	*MAT* **a** lys2 *lys2* *trp1*::hisG *ura3* *LYS2*::ho	This study
RSY877	*MAT* **a**/*MAT*α lys2 *lys2* *trp1*::hisG *ura3* *LYS2*::ho	This study
RSY1823	*MAT* **a** *lys2* *trp1*::hisG *ura3* *LYS2*::ho *rpl22A*::KANMX *rpl22B*::KANMX	This study
RSY1826	*MAT* **a** *lys2* *trp1*::hisG *ura3* *LYS2*::ho *rpl22A*::KANMX *rpl22B*::KANMX	This study
RSY1997	*MAT* **a**/*MAT*α *cyh2* ^R^-*z* *ho*::*LYS2* *leu2*::hisG *lys2* *trp1*::hisG *ura3* *rpl39*::KANMX	This study
RSY2003	*MAT* **a**/*MAT*α *cyh2* ^R^-*z* *ho*::*LYS2* *leu2*::hisG *lys2* *trp1*::hisG *ura3* *rpl29*::KANMX	This study

^a^All markers shown are homozygous in the *MAT* **a**/*MAT*α strains unless indicated

### Media and phenotypic assays

Cultures were grown in rich YPDA (2 % dextrose, 2 % peptone, 1 % yeast extract supplemented with 10 mg/l adenine). Plasmid selection was maintained in strains using synthetic dextrose (SD) medium containing 0.17 % yeast nitrogen base without amino acids, 0.5 % ammonium sulfate, 2 % dextrose. Pre-sporulation growth was conducted in either YPA (K acetate (1 %) substituted for dextrose in YPDA) or synthetic acetate (SA, 0.17 % yeast nitrogen base without amino acids, 0.5 % ammonium sulfate, 2 % K acetate). Liquid sporulation medium (SPM, 2 % K acetate supplemented with uracil) was utilized for meiotic timecourse experiments. Invasive growth assays were performed by streaking cells on to YPDA agar plates, incubated for 3 days at 30 °C then washed with a gentle stream of water, while clearing cells on the surface with a gloved hand [3]. Pseudohyphal growth was assayed by streaking wild type or *rpl22∆* diploid SK1 cells for single colonies onto synthetic, low-ammonia, dextrose (SLAD) agar plates followed by incubation for 5 days at 30 °C [4, 7]. Meiotic timecourse experiments were conducted with cells grown to mid-log phase in YPA or synthetic acetate (SA), washed in water, and resuspended in sporulation medium (SPM) as previously described [27]. Nuclear divisions were monitored by fixing cells with 70 % ethanol at 4°, washed twice with water, then stained for 15 min with 1 μg/ml 4′,6′-diamidino-2′-phenylindole (DAPI). The cells were washed twice with water and visualized by fluorescence microscopy.

### Protein extraction, western blotting, cycloheximide chase assay

Approximately 5 × 10^7^ cells were treated with 0.2 M sodium hydroxide, with subsequent extraction in Laemilli buffer accompanied by glass bead lysis [28]. 1 × 10^7^ cell equivalents were loaded for each sample. Proteins were separated by 10 % SDS-PAGE, transferred to PVDF membranes and blots were probed with anti-HA (12CA5, Roche), anti-Tub1p (Developmental Studies Hybridoma Bank, University of Iowa), poly-clonal anti-Ume6 or anti-Pgk1p (Invitrogen) monoclonal antibodies and visualized using AP conjugated anti-mouse secondary antibody and the CDP-Star system. Cycloheximide (CHX) chase assays were performed essentially as described [29].

### In vivo translation analysis

Exponentially growing cells in rich media were depleted of their methionine and cysteine stores through growth in defined medium lacking these amino acids. After an hour of incubation, 125 μCi of ^35^S labeled methionine and cysteine were introduced to the medium and incubated at 30 °C. Samples were taken, washed, and frozen in liquid nitrogen every 5 min for 20 min. The proteins were extracted in Laemmli buffer and 1 × 10^7^ cell equivalents were either precipitated using methanol and chloroform to remove unincorporated label to measure total isotope incorporation, or run on a polyacrylamide gel for radiography.

### Polysome profiles

Polysome profiles were performed for each of the given nutritional conditions, as described [30]. Cultures were treated with 100 μg/ml (final concentration) cycloheximide. Harvested cells were washed in lysis buffer in the presence of cycloheximide and heparin, and lysed using glass beads at 4º C. Lysates were clarified with sequential centrifugation (5K×*g*, 5 min; 13K×*g*, 10 min) and approximately 200 μg of total RNA was loaded on 15–50 % sucrose gradients. Gradients were centrifuged for 4.25 h at 160K×*g*. Gradients were analyzed using a continuous flow cuvette. For mRNA analysis of polysomes, wild-type and *rpl22Δ* cells were grown in 50 ml of YPA and shifted to 10 ml of sporulation medium for 9 h. A small sample was taken for total RNA with the remaining cells treated with cycloheximide (100 μg/ml for 5 min), crosslinked (1 % formaldehyde for 5 min), then the crosslinking quenched with glycine (250 mM). The cells were harvested by centrifugation and snap frozen in liquid nitrogen. Lysates were prepared, centrifuged through a sucrose density gradient, and fractionated as previously described [31]. Fractions were treated with 1 % SDS, 16.6 mM EDTA, and 0.1 mg/ml proteinase K, and incubated at 42 °C for 1 h, then 65 °C for 1 h to reverse crosslinks. Fractions were then extracted with an equal volume of phenol–chloroform–isoamyl alcohol (25:24:1), and precipitated with an equal volume of isopropyl alcohol. The RNA pellets recovered were washed twice in 70 % ethanol, and equal proportions of each fraction were analyzed by Northern blot. Five microgram each of total RNA and crude lysate were loaded alongside RNA recovered from the sucrose density gradient. Membranes were probed for *IME1* and *ENO1* as described above, and imaged on a Typhoon Phosphor Imager (GE Healthcare).

### Northern blotting and qRT-PCR

Frozen cell pellets from meiotic time courses were lysed with glass beads and phenol–chloroform extracted as previously described [6]. RNA pellets were resuspended in DEPC treated dH_2_O, quantitated, and equal masses were run on 1.2 % agarose formaldehyde gels. Separated RNA was blotted using capillary action and probed for the genes of interest indicated. Probes used were gel purified PCR products or restriction digests of the ORF of the genes of interest and randomly labeled using Klenow and α-^32^P labeled dCTP. qRT-PCR was performed with similarly extracted RNA. RNA was reverse transcribed using AMV-RT (NEB), and amplified using primer pairs of the genes of interest using Power Sybr (AppliedBiosystems), listed in Table 2.

**Table 2:** qRT-PCR primers used in this study

Primer	Name sequence
ENO1-F	GCC GCT GCT GAA AAG AAT GT
ENO1-R	TGG AGA GGT CTT GGA CTT AGA CAA
IME2-F	AAT GTT TTG GGT GAT GCC TCT T
IME2-R	TTC TTG GAG TAA AAT CTG GCA TTG
NUP85-F	TTC GCG AAG GAG CAT AAT GC
NUP85-R	ACA CTT CCA ATT CAT TCA GAA TCG
RPL22A-F	CCA AGA CCT TTA CCG TCG ATG T
RPL22A-R	AGG AAG CTG GGT CGA AGA C
RPL22B-F	GAG TCT TCG ATC CGG CTT CA
RPL22B-R	TTC CTA CGG CAC CAT CTA CTT TAA T

25S:18S rRNA ratios were determined for logarithmic cultures or 9 h following the shift to SPM. Total RNA dilutions (2, 1, or 0.5 μg) from each condition were analyzed by Northern blot probing with the Y503 and Y500 rRNA probes as described [31]. The signals were quantitated by phosphorimager and the 25S:18S ratios calculated for each condition. Ratios were averaged for each condition and normalized to the wild-type rich growth (YPD) value. The relative error was propagated from the standard deviation obtained from each averaged ratio.

## Results

### Rpl22 is a non-essential ribosomal protein

**Fig. 1:**
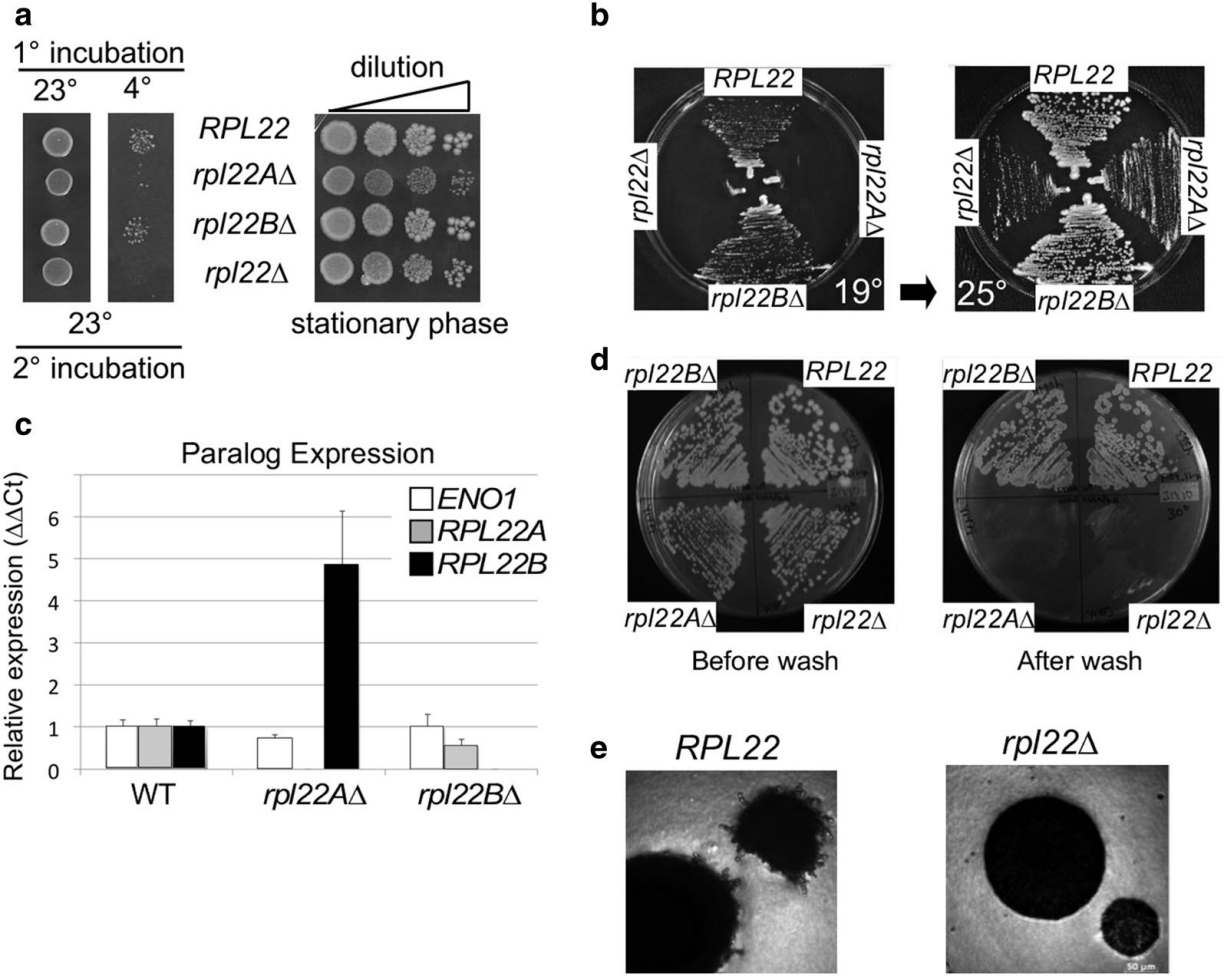
Rpl22 is a nonessential protein required for hyphal growth. **a** Wild type (RSY333), *rpl22AΔ* (RSY1479), *rpl22BΔ* (RSY1446) and *rpl22Δ* double mutant (RSY1483) cultures were incubated at the indicated temperatures for 24 h in rich (YPD) liquid culture then spotted onto rich agar plates and incubated at 23° (*left panel*). The same cultures were grown in rich medium at 23° for 5 days, then a dilution series was spotted on rich agar and incubated at 23° for 2 days. **b** The strains described in **a** were streaked on YPD agar plates and incubated at 19 °C for 4 days (*left panel*), then 30 °C for 2 days (*right panel*). **c** Wild type (RSY333), *rpl22AΔ* (RSY1479) and *rpl22BΔ* (RSY1446) cultures were grown to mid-log and total RNA preparations were subjected to qRT-PCR analysis. *RPL22A* and *RPL22B* mRNA levels were normalized to *NUP85* in wild type then analyzed in each single mutant. *ENO1* mRNA levels were quantified as a RNA concentration control. The results are referenced to wild type levels ± SEM (n = 3). **d** Haploid strains described in **a** were streaked on rich growth medium for 2 days (*left panel*), then subjected to a stream of water, the re-incubated for 24 h (*right panel*). **e** SK1 derived diploid wild type (RSY877) and *rpl22Δ* (RSY1823) double mutant cells were assayed for pseudohyphal growth by microscopy (×60 final magnification)

In both zebra fish and mice, L22 is not essential for the production of adult animals [24]. However, defects in specific developmental pathways were identified. To assess what role, if any, Rpl22 played in differentiation processes in yeast, either single (*rpl22A∆* or *rpl22B∆*) or double (*rpl22∆*) strains were constructed. Similar to a previous report [18], Rpl22A and/or Rpl22B function is not required for mitotic cell division in rich medium although the doubling times for the *rpl22A∆* and *rpl22∆* double mutant strains were slightly reduced compared to wild type or *rpl22B∆* cultures (data not shown). However, we found that *rpl22A∆* or *rpl22∆* strains were unable to form colonies when incubated at 4 °C for 24 h then returned to growth at 25° (Fig. 1a). To determine whether this phenotype was due to the reduced temperature itself, or the result of a return to growth defect, cultures were incubated at 25° for 3 days to arrest cell division in stationary phase. The cells were diluted then replated on rich medium and incubated at 25°. This experiment found no difference in the return to growth of any mutant compared to wild type (Fig. 1a, right panel). These results indicate that Rpl22A is required for survival at low temperatures.

To examine this phenotype further, wild type, *rpl22A∆*, *rpl22B∆* and *rpl22∆* double mutants were streaked on rich growth medium and incubated at the intermediate temperature of 19 °C for 4 days. The wild type and *rpl22B∆* strains grew similarly while the *rpl22A∆* and double mutant failed to form colonies (Fig. 1b, left panel). To determine whether these strains were growth arrested or lost viability, the same plates were then placed at 25 °C for 2 days and images obtained. In this experiment, growth was observed for the *rpl22A∆* and double mutant (right panel) indicating that a mild cold shock is sufficient to arrest cell division but not kill the cell while a more severe reduction in temperature results in cell death.

The phenotypic differences observed between *rpl22A∆* and *rpl22B∆* mutants may be explained by the finding that *RPL22A* is transcribed at much higher levels than *RPL22B* [32]. Another contributing factor could also be the regulation of one paralog by the other [33]. Therefore, we examined mRNA levels of each gene in logarithmic cultures by qRT-PCR. *RPL22A* and *RPL22B* mRNA levels were first standardized to control transcripts (*NUP85* and *ENO1)*. Our control genes were expressed at relatively comparable levels, regardless of the gene deletion (Fig. 1c). In the absence of *RPL22A*, *RPL22B* expression increased fourfold while deletion of *RPL22B* did not affect *RPL22A* mRNA levels. These results indicate that loss of one *RPL22* allele does not adversely impact the activity of the other. These findings are consistent with the model that elevated Rpl22A expression levels represent a major cause for our observed phenotypic differences.

### Rpl22 is required for invasive and pseudohyphal growth

As described above, Rpl22 regulates metazoan cell differentiation pathways. Similarly, yeast exhibit several alternative cell fates controlled by cell type and environmental cues. A nitrogen-diminished environment triggers changes in cell cycle and cell shape in haploid or diploid cells termed invasive or pseudohyphal growth, respectively [7]. Therefore, we investigated a role for *RPL22* in these processes. First, we determined whether Rpl22A and/or Rpl22B were required for invasive growth. Haploid wild-type, *rpl22AΔ*, *rpl22BΔ*, and *rpl22Δ* double mutant cells were grown on rich solid medium then the plate was washed with water. Cells embedded in the agar due to invasive growth will be resistant to washing. Cells lacking *RPL22A* or both paralogs were unable to significantly penetrate the agar, while wild type and *rpl22BΔ* cells were embedded (Fig. 1d). These results indicate that Rpl22A is required for invasive growth. Next, we tested the requirement of Rpl22 for pseudohyphal growth by generating diploid strains lacking both *RPL22* paralogs in the SK1 strain background. This background was chosen as it exhibits a robust pseudohyphal growth phenotype. Wild type and *rpl22Δ* double mutant cells were streaked on SLAD plates which contain limiting nitrogen, incubated at 30 °C for 4 days. Microscopic examination revealed a radial growth from the center of in wild-type colonies. Conversely, no *rpl22Δ* cells exhibited this phenotype (500 cells examined, Fig. 1e). These results indicate that Rpl22 plays a second role in the switch from budding to hyphal forms of cell division. Taken together, these results indicate that Rpl22 plays an important role in the morphogenic switches that respond to a reduced nitrogen environment.

### Rpl22 is required for the execution of meiotic development

**Fig. 2:**
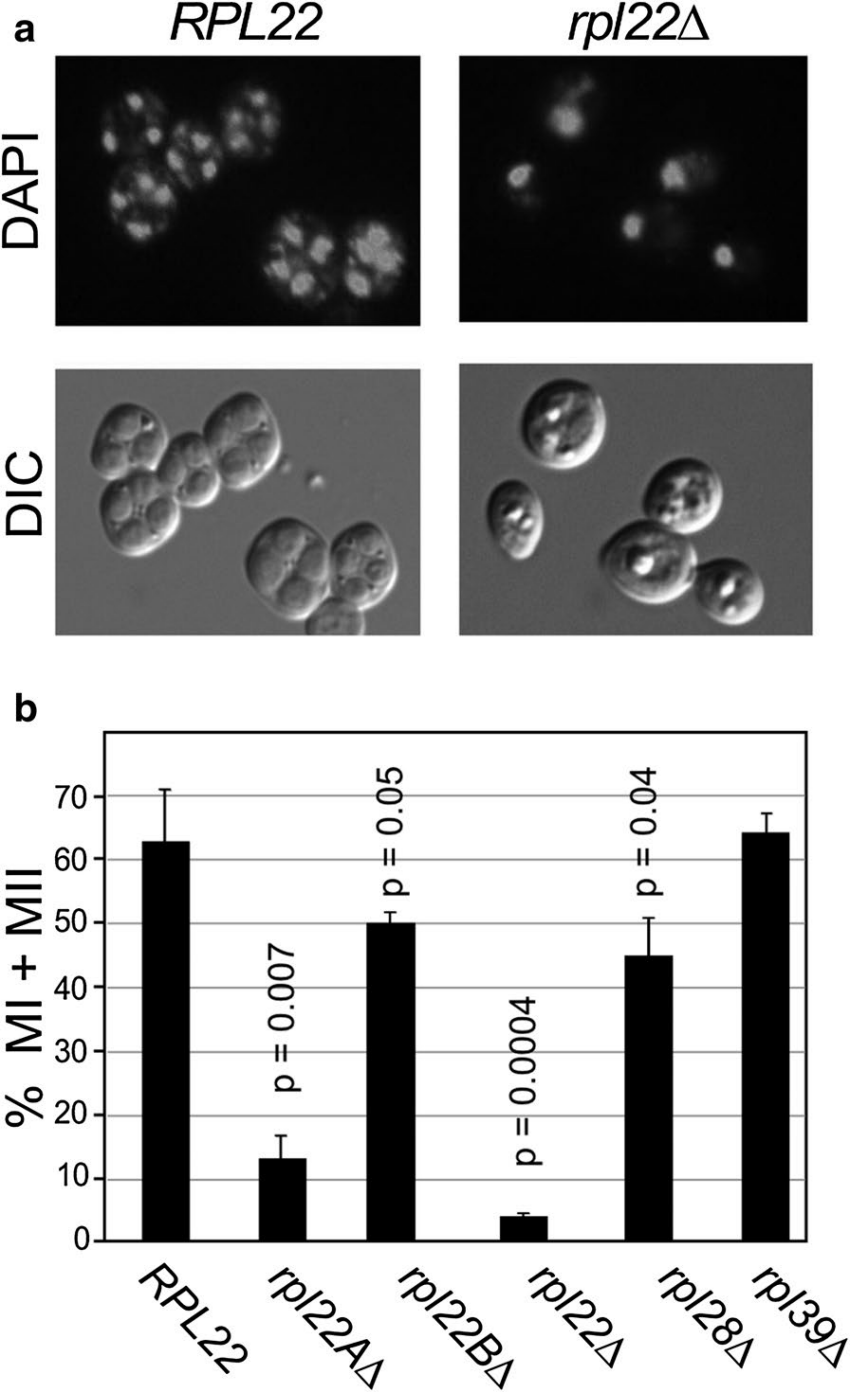
Rpl22 is required for execution of meiotic nuclear divisions. **a** Wild type (RSY335) and *rpl22Δ* double mutant (RSY1561) cultures were grown in rich acetate (YPA) then shifted to sporulation medium (SPM) for 24 h and monitored for the production of bi-nucleated and tetranucleated cells by fluorescence microscopy of DAPI stained cells. **b** The percent of bi- or tetra-nucleated cells in the population is shown for the strains described in **a** and *rpl22AΔ* (RSY1559), *rpl22BΔ* (RSY1560) *rpl28∆* (RSY2003) and *rpl39∆* (RSY1997). *Error bars* equal SEM p values compared to wild type are indicated when significant differences were observed (n ≥ 3)

Similar to filamentous growth, entry into meiosis is controlled by both cell type and exogenous signals. To test whether Rpl22 is required for meiosis and subsequent spore formation, wild type, *rpl22A∆*, *rpl22B∆* and *rpl22Δ* diploids were grown to mid-log phase in rich growth medium containing the non-fermentable carbon source acetate (YPA). These cultures were harvested, washed, then transferred to sporulation medium (SPM) and time points taken. The percentage of each population able to undergo either one or both meiotic nuclear divisions was determined by DAPI staining and fluorescence microscopy. In this experiment, meiotic nuclear divisions began by 12 h following transfer to SPM in the wild-type culture reaching ~60 % by 24 h (Fig. 2a, quantitated in Fig. 2b). The *rpl22A∆* strain exhibited a significant reduction (p = 0.001) in bi- and tetra-nucleated cells compared to wild type while *rpl22B∆* cells showed a modest but significant (p = 0.05) loss in cells executing one or both meiotic divisions. However, only 2 % of *rpl22Δ* double mutant cells were able to complete either round of division. These results indicate that Rpl22 is required for execution of the meiotic nuclear divisions.

To determine if this meiotic role for Rpl22 was a general property of non-essential ribosomal proteins, two additional large subunit proteins, Rpl39 and Rpl29, were analyzed. Rpl29 localizes to the 40S interaction face and is required for efficient subunit association [34]. Conversely, Rpl39 resides near the peptide exit channel [35]. Finally, these nonessential RP genes were chosen as their deletion reduced growth rates to a level similar to *rpl22A∆* mutants [18]. Unlike *RPL22*, *RPL39* and *RPL29* are not duplicated so only single *rpl39∆* or *rpl29Δ* diploids were constructed. Their ability to undergo meiosis was assessed as just described. These experiments revealed a modest but significant (p = 0.03, n = 3–5) difference between wild type and the *rpl39∆* diploid while no difference was observed in *rpl29∆* cells (Fig. 2b). However, the eightfold difference between *rpl39∆* and *rpl22∆* mutant culture meiotic efficiencies indicates that a severe meiotic defect is not a general feature of deleting non-essential ribosomal genes.

### Rpl22 is required for IME1 mRNA translation

**Fig. 3:**
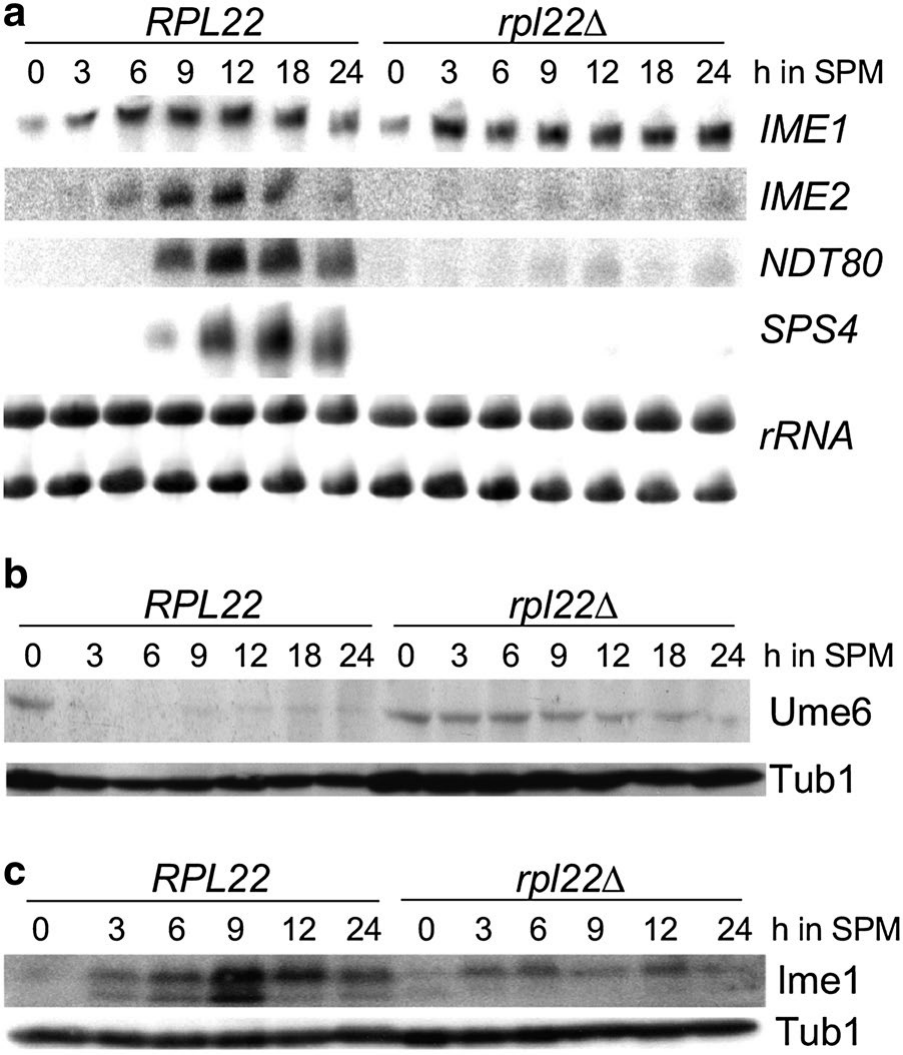
Rpl22 is required for meiotic accumulation of Ime1. **a** Wild type (RSY333) and *rpl22Δ* (RSY1561) double mutant cells were grown to mid-log phase in YPA and shifted to SPM and samples taken at the times indicated. Total RNA preparations were subjected to Northern blot analysis and probed for the *IME1*, early (*IME2*), early–middle (*NDT80*), and middle (*SPS4*) meiotic genes as indicated. Ethidium bromide stained rRNA served as the loading control. **b** Western blot analysis of protein extracts prepared from the same meiotic timecourse described in A was probed for endogenous Ume6. Tub1 levels served as a loading control. **c** Wild type (RSY1833) and *rpl22Δ* (RSY1839) double mutant diploids expressing *IME1*-3HA were subjected to a meiotic timecourse experiment. Protein extracts prepared from the indicated timepoints were probed for the presence of Ime1-3HA. The blots were reprobed for Tub1 which served as a loading control

The inability of *rpl22∆* double mutants to undergo meiosis I or meiosis II could be due to a failure to exit the mitotic cell cycle or represent an early meiotic arrest prior to anaphase I. To address this question, wild type and *rpl22∆* double mutant were induced to enter meiosis and Northern blot analysis was performed on total RNA prepared from different time points. The resulting blots were probed for mRNAs transcribed at different stages of meiotic development. In the wild-type culture, a normal transcription profile was observed with the transient transcription of meiotic inducer *IME1*, as well as *IME2*, *NDT80*, and *SPS4,* members of the “early”, “early-middle” and “middle” expression classes, respectively (Fig. 3a). However, although *rpl22Δ* cells robustly expressed *IME1* mRNA, the levels of *IME2*, *NDT80*, and *SPS4* transcripts were at or below the limits of detection. These results indicate that *rpl22∆* mutants enter the meiotic program but arrest prior to the transcription of the early meiotic gene class.

Early meiotic gene induction requires the destruction of the transcriptional repressor Ume6 [6, 36]. The lack of *IME2* transcript accumulation suggested that the Ume6 repressor is not destroyed in the *rpl22∆* mutant. To test this model, wild type and *rpl22∆* double mutant diploids were subjected to a meiotic timecourse experiment and endogenous Ume6 levels were monitored by Western blot analysis of total protein extracts. As observed previously [6], Ume6 levels are reduced below the limits of detection in the wild-type culture shortly after transfer to SPM (Fig. 3b). However, Ume6 levels remained constant in the *rpl22∆* mutant strain until late in the timecourse. These results indicate that Rpl22 is required for Ume6 destruction and subsequent meiotic progression.

We previously reported that Ime1 association is required for the APC/C^Cdc20^ ubiquitin ligase-directed proteolysis of Ume6 [6, 36]. Therefore, we next examined Ime1 levels during meiosis in a wild type and *rpl22∆* double mutant strains. The *IME1* allele was chromosomally tagged with three copies of the hemagglutinin (3HA) epitope to allow Ime1 detection by Western blot analysis. Sporulation kinetics and efficiency were indistinguishable between the wild-type strain expressing Ime1 or Ime1-3HA indicating that the tagged allele is functional (data not shown). In the wild-type strain, the Ime1-3HA signal was detected by 3 h following transfer to SPM with peak expression occurring at 9 h (Fig. 3c). In the *rpl22∆* cells, Ime1-3HA was detected at 3 h but its levels remained flat throughout the timecourse and did not exhibit a spike in expression. These results suggest that Rpl22 is required for normal Ime1 accumulation, which in turn leads to Ume6 destruction and meiotic progression.

### Rpl22 is required for efficient *IME1* mRNA translation

**Fig. 4:**
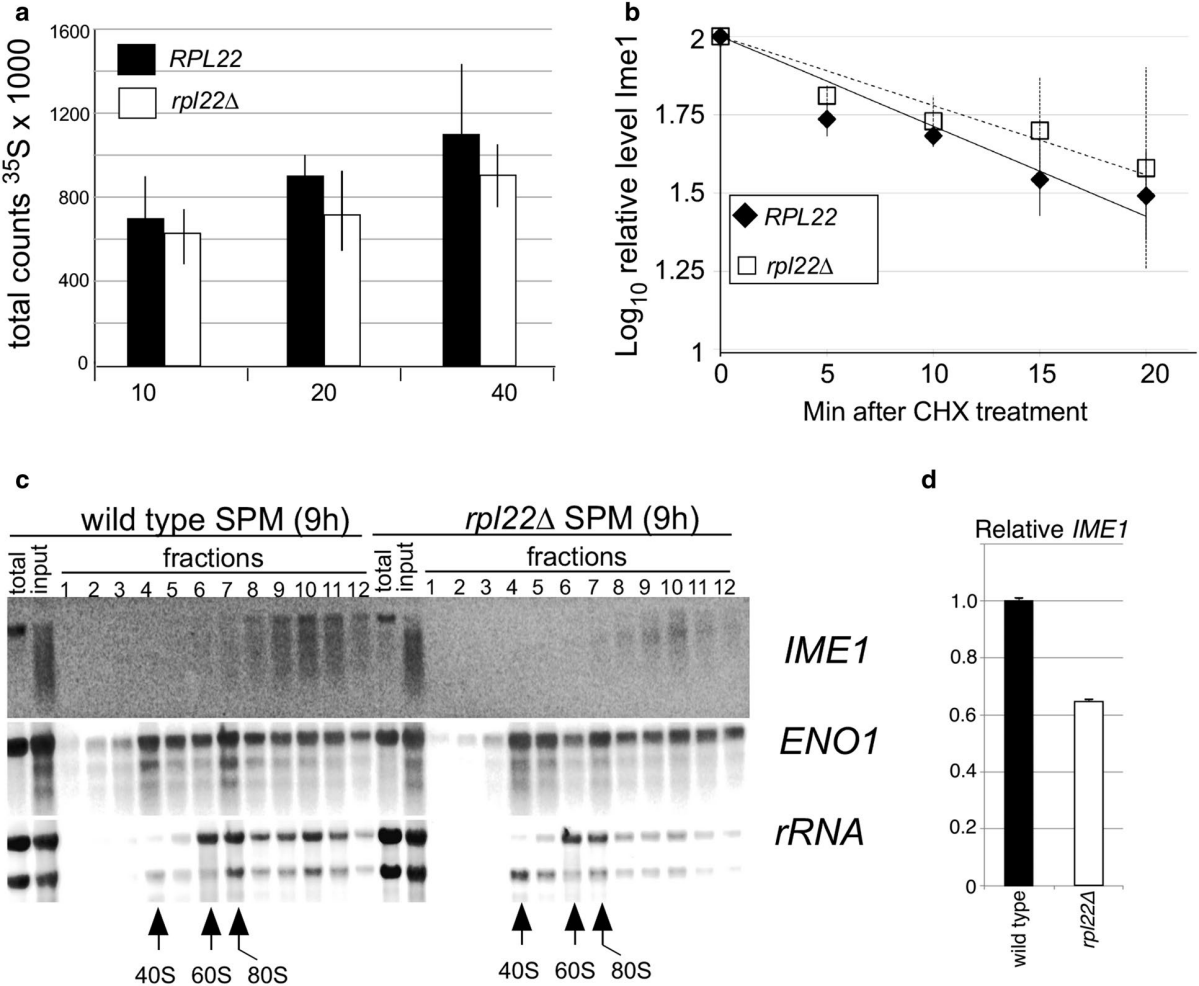
Rpl22 enhances *IME1* mRNA translation. **a** Incorporation of ^35^S methionine and cysteine into bulk protein was monitored by scintillation spectroscopy in wild type (RSY333) and *rpl22∆* double mutant (RSY1483) at the indicated times (min) following addition of radioactive amino acids to starved cultures. *Error bars* represent SEM from two experiments with two independent isolates each. **b** Wild type (RSY333) and *rpl22Δ* (RSY1561) double mutant cells were induced to enter meiosis and cycloheximide was added at 9 h following transfer to SPM. Timepoints were taken after addition of CHX at the times indicated. Ime1-3HA specific chemiluminescence signals were detected by Western blot analysis and quantified by CCD camera. Graph shows the averaged results from two independent experiments. *Error bars* indicate SEM. **c** Polysomes were isolated from wild type and *rpl22∆* double mutant cultures 9 h following transfer to SPM. Total RNA isolated from the indicated fractions was subjected to Northern blot analysis with the indicated probes. The position of the ribosomal subunits and monosomes are indicated. Total RNA lane indicates samples prepared directly from harvested cells. Input represents total RNA isolated from polysome extracts prior to fractionation. **d** The relative amounts of *IME1* mRNA in total RNA preparations from wild type (RSY333) and *rpl22Δ* (RSY1561) double mutant cells were determined by qRT-PCR. The graph shows the results and SEM from three technical replicates from one experiment

Our results indicate that Rpl22 is required for Ime1 accumulation in meiotic cells. There are several mechanisms that could explain this result. First, Rpl22 may have a general impact on translation. To test this possibility, translation by quantified by measuring ^35^S-Met and ^35^S-Cys incorporation. Isolates of wild type and *rpl22Δ* cells were starved of methionine and cysteine for an hour. ^35^S labeled methionine and cysteine were added to the medium and samples were collected at the times indicated (Fig. 4a). Extracted proteins were precipitated and the radioactivity counted by scintillation spectroscopy. By 10 min following addition of the labeled amino acids, a small reduction in ^35^S incorporation was observed in *rpl22∆* double mutants compared to wild type that continued throughout the timecourse. These differences were not significant and indicate, consistent with the modest reduction in growth rates, that Rpl22 is not required for bulk translation. Next, we tested whether Rpl22 is required to maintain Ime1 protein stability. To examine this question, Ime1 turnover was monitored using cycloheximide (CHX) translation shut off experiments. CHX was added to wild-type and *rpl22∆* diploid cultures 9 h following transfer to SPM. Timepoints were taken and protein extracts prepared from these samples were subjected to Western blot analysis. Quantitation of the Ime1 signal revealed a similar decline in protein levels (Fig. 4b). These results indicate that Rpl22 does not control Ime1 turnover.

Our results indicate that Rpl22 does not control *IME1* transcription or Ime1 protein stability. These findings point to translation as a potential explanation for reduced Ime1 accumulation in the *rpl22∆* strains. Translation defects can be due to failure of the mRNA to successfully exit the nucleus and associate with the ribosome or defects in the translation process itself. To test these possibilities, we probed for the presence of *IME1* mRNA in ribosome fractions. Cultures taken 9 h following transfer to SPM and treated with a combination of CHX and formaldehyde to stall and crosslink ribosomes to mRNA. This arrest protocol was employed as we discovered that conventional CHX translation arrest resulted in severe *IME1* mRNA degradation (data not shown). Total RNA isolated from these polysomes was subjected to Northern blot analysis probing for *IME1* mRNA. These studies revealed that *IME1* mRNA was still degraded in both wild type and *rpl22∆* diploids when compared to other control transcripts (*ENO1* or rRNA). However, *IME1* mRNA was still detected in both wild type and *rpl22∆* mutant polysome fractions although the levels appeared reduced in the mutant fractions (Fig. 4c). Quantitating the mRNA samples did reveal that *IME1* mRNA levels were reduced approximately 40 % in the *rpl22∆* sample in this experiment (Fig. 4d). Taking this result into consideration, this experiment indicates that *IME1* mRNA is associated with polysomes in meiotic *rpl22∆* cells. These findings suggest that Rpl22 functions following ribosome binding but prior to translation initiation (see “Discussion” section). In addition, these results suggest that *IME1* mRNA maybe specifically targeted for degradation on stalled ribosomes.

### Rpl22 operates through the *IME1* 5′UTR to promote translation

**Fig. 5:**
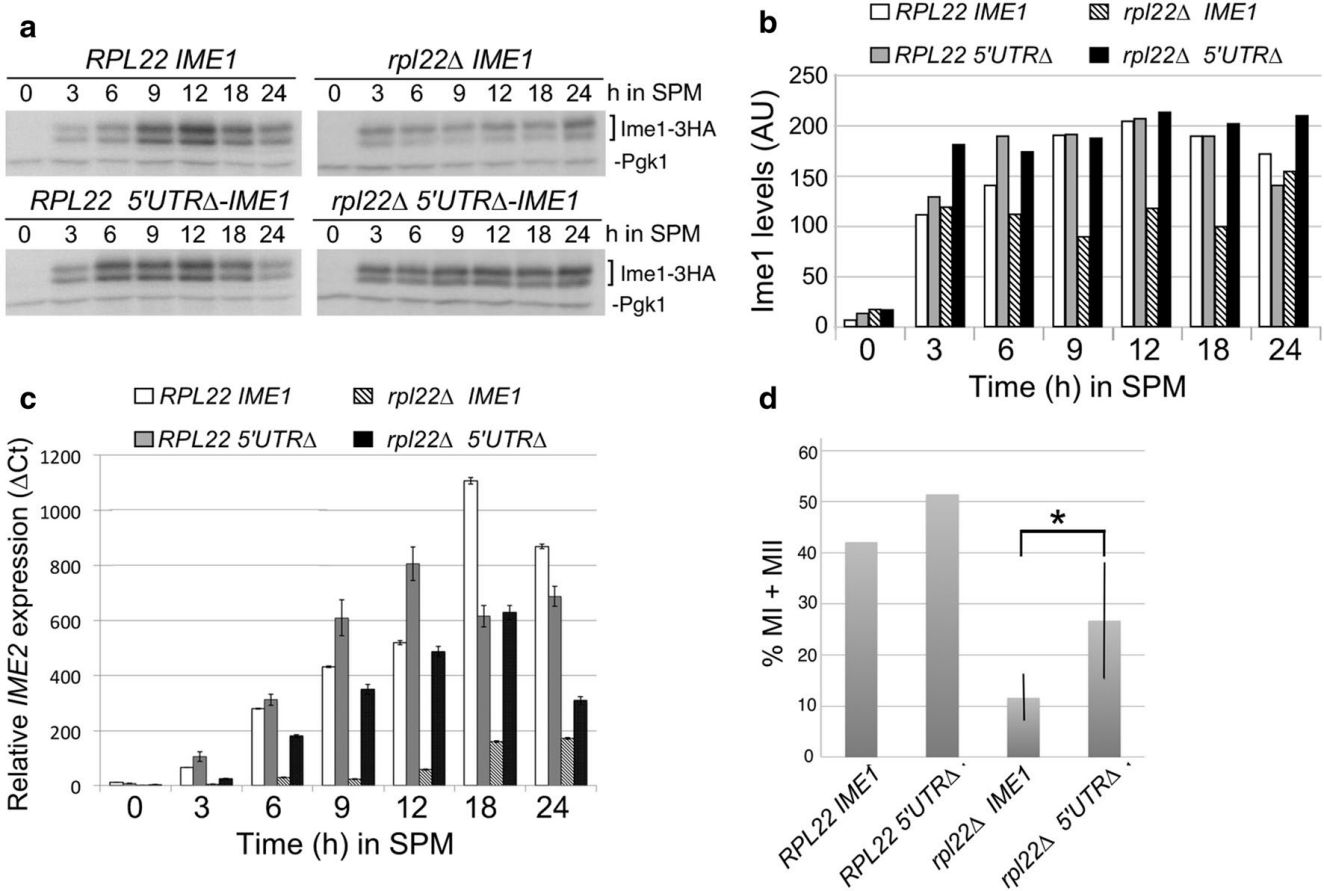
Rpl22 supports translation through the *IME1* 5′UTR. **a** Western blot analysis of protein samples prepared from wild type (RSY1833, RSY1991) and *rpl22Δ* double mutant (RSY1839, RSY1993) strains harboring *IME1*-3HA or 5′UTRΔ-*IME1* alleles, respectively. The blots were probed for Ime1-3HA and Pgk1, which served as a loading control. **b** The Ime1 signals obtained in **a** were quantified, normalized to Pgk1 levels, and plotted for each timepoint (see “Methods” section for details). **c** *IME2* mRNA levels were determined by qRT-PCR during a meiotic timecourse with the strains described in **a**. The results depicted are the average from three technical replicates from one experiment. **d** Terminal sporulation efficiencies following 24 h in SPM were determined for the strains described in **a**. The values for *rpl22Δ* and 5′UTRΔ-*IME1* are the result of two trials of three independent cultures. *Error bars* indicate SEM. *Asterisk* indicates p = 0.05

We next sought an underlying mechanism to explain the role of Rpl22 in *IME1* mRNA translation. *IME1* mRNA is unusual among yeast transcripts for containing a large 230 nt 5′UTR, which has been hypothesized to regulate translation [10]. We hypothesized that the long 5′UTR may make *IME1* mRNA refractory to translation by ribosomes lacking Rpl22. To test this possibility, we genomically deleted 180 nt of the *IME1*-*5′*UTR (5′UTR∆-*IME1*) in wild-type and *rpl22Δ* double mutant cells. This deletion was selected as it maintained the local context of both the transcriptional and translational start sites. We monitored Ime1-3HA protein levels from the wild type or 5′UTR∆-*IME1* allele during a meiotic time course experiment. As before (Fig. 3c), Ime1 levels were induced at the same time but failed to display stage-specific induction in the *rpl22∆* strain compared to wild type (top panels, Fig. 5a, quantitated in Fig. 5b). Deleting the 5′UTR lead to earlier Ime1 induction compared to wild type (see 6 h timepoint, left panels) but both strains exhibited reduced Ime1 levels by 24 h. These results suggest that that the 5′UTR is not important for neither the initial accumulation of Ime1 nor its down regulation later in development. In the *rpl22∆* 5′UTR∆-*IME1* strain, Ime1 was fully induced by the 3-h timepoint and remained elevated throughout the experiment. These results are consistent with a model that the 5′UTR inhibits *IME1* mRNA translation that is relieved by Rpl22 function. In addition, these results suggest that Rpl22 plays a role in re-establishing repression of Ime1 levels as the culture completes the meiotic program.

Next, we determined whether relieving the 5′UTR block to translation was sufficient to restore Ime1 function and complete meiosis in the *rpl22∆* mutant. First, as an indicator of Ume6 destruction, *IME2* mRNA expression was compared in an *rpl22∆* double mutant diploid harboring either wild type *IME1* or the 5′UTR∆-*IME1* deletion allele. Total RNA was prepared from a meiotic timecourse experiment and *IME2* mRNA concentrations were determined by qRT-PCR. As previously described, *IME2* mRNA was not induced in *rpl22Δ* cells containing the intact *IME1* 5′UTR (hatched box, Fig. 5c). Although delayed by a timepoint, *IME2* mRNA was induced to levels higher in the *rpl22∆* 5′UTR∆-*IME1* mutant (black box) compared to *rpl22∆ IME1* cells. These results indicate that the increase in Ime1 observed in the 5′UTR∆-*IME1* strain was sufficient to induce Ume6 destruction and subsequent *IME2* transcription but not quite to wild-type levels. Interestingly, although Ime1 levels were induced early and stayed elevated in the *rpl22∆* 5′UTR∆-*IME1* cells, the kinetics of *IME2* mRNA accumulation were slower than wild type. These results suggest that Rpl22 has a role in *IME2* mRNA induction in addition to *IME1* mRNA translation.

We next tested whether deletion of the 5′UTR could rescue the *rpl22Δ* sporulation phenotype. In the *RPL22* strain, the presence of the 5′UTR∆ allele did not alter sporulation efficiency compared to the intact *IME1* allele (Fig. 5d) indicating that the differences in Ime1 expression kinetics do not affect the efficiency of meiotic divisions. In the *rpl22∆* double mutant, the presence of 5′UTR∆-*IME1* allowed a significant increase in sporulation efficiency compared to *rpl22∆* cells harboring wild type *IME1*. These results indicate that deleting the 5′UTR can bypass the meiotic defect in a *rpl22∆* double mutant. However, the rescue was not to the levels observed in wild type cells. This observation, combined with the *IME2* mRNA analysis in the *rpl22∆* 5′UTR∆-*IME1* strain, suggests that Rpl22 has additional execution points later in meiosis (see “Discussion” section).

### Rpl22 is required for normal polysome assembly

**Fig. 6:**
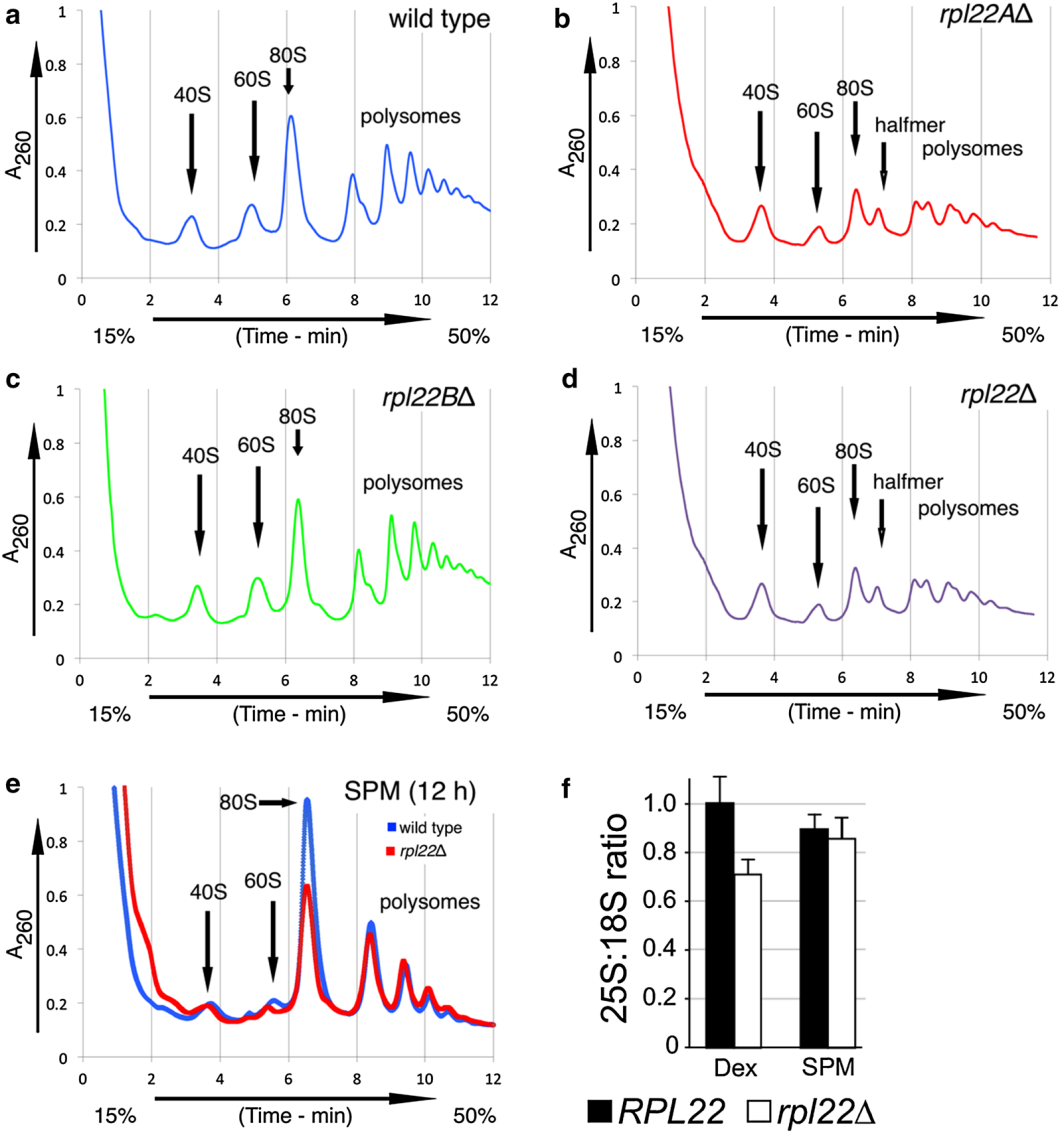
Rpl22 is required for normal polysome profiles under conditions of high translational output. **a** Logarithmically growing wild type (RSY335), **b** *rpl22AΔ* (RSY1559), **c** *rpl22BΔ* (RSY1560) and **d** *rpl22Δ* double mutant (RSY1561) cells in rich dextrose medium (YPD) were subjected to polysome profile analysis through sucrose density gradient centrifugation. The *arrows* indicate the 40S, 60S, 80S monosome and halfmer positions. **e** Overlaid polysome profiles for wild type and *rpl22∆* strains taken 9 h following transfer to SPM medium. **f** The 25S:18S rRNA ratios were quantified by Northern blot analysis for wild type and the *rpl22∆* double mutant in log phase cells grown in rich dextrose (YPD) medium. The cumulative differences between the 25S:18S ratios in wild type and *rpl22∆* double mutant strains were significantly different (p = 0.02) in YPD but not SPM

Our results indicate that Rpl22 is required for normal *IME1* mRNA translation even though the transcript is found in the polysomes. Therefore, we next determined whether Rpl22 is required for normal polysome assembly. Velocity sedimentation gradients were utilized to generate polysome profiles of logarithmically growing wild type and *rpl22* mutant cells in rich dextrose medium. The wild-type profile exhibited the expected monosome (80S) and polysome peaks, as well as free small (40S) and large (60S) subunits (Fig. 6a). A similar profile was observed for *rpl22B∆* mutant extracts (Fig. 6c). However, several differences in the polysome profile were observed in *rpl22A∆* or the *rpl22∆* double mutant strains (Fig. 6b, d, respectively). First, we found that *rpl22Δ* or *rpl22A∆* cells exhibited reduced 60S:40S particle ratio compared to wild type (quantified in Fig. 6f). In addition, these profiles exhibited doublets at the 80S peak and each subsequent peak in the polysome fraction. These peaks are indicative of “halfmer” formation that is usually diagnostic for a bound 40S particle that is not stably bound by the 60S subunit [37–39]. Taken together, these results indicate that Rpl22 plays an important role in 60S subunit assembly and/or its ability to stably associate with the 40S particle.

We hypothesized that defects in polysome formation may explain the observed lack of Ime1 accumulation. Therefore, we performed polysome analysis on wild-type and *rpl22Δ* cells 12 h after shifting to sporulation medium (Fig. 6e). Interestingly, meiotic *rpl22Δ* cells did not exhibit the halfmer phenotype as the free 40S and 60S subunit peaks in both sporulating wild type or *rpl22Δ* cells were largely absent. One possibility is that idle ribosomal subunits are catabolized upon entry into meiosis, relieving “halfmer” formation in polysome peaks. Since normal profiles were obtained from meiotic *rpl22∆* cells, these results suggest that the halfmer formation and the failure to translate *IME1* mRNA represent separate phenotypes associated with loss of Rpl22 function.

## Discussion

Changes in cell fate require remodeling the gene expression program at the level of both transcription and translation [12, 40]. Although transcriptional control has been the focus of extensive study, it is becoming increasing clear that regulated translation also mediates these decisions. In this report, we demonstrate that the non-essential large subunit ribosomal protein Rpl22 is required for the developmental switch from normal mitotic cell division to either invasive/pseudohyphal growth or meiotic entry. As meiosis and hyphal growth are induced under conditions of limiting or depleted environmental nitrogen, Rpl22 may represent a mediator of low-nitrogen dependent translation. To promote meiotic induction, Rpl22 is necessary for efficient translation of the meiotic inducer *IME1* mRNA. Importantly, the requirement of Rpl22 for *IME1* mRNA translation can be suppressed by deleting the unusually long *IME1* 5′UTR. Formally, these results indicate that Rpl22 operates through this region. However, only partial restoration of sporulation efficiency was observed in the *rpl22∆* mutant expressing *IME1* lacking the 5′UTR suggesting that additional execution points for Rpl22 exist during meiosis. Taken together, these results suggest that Rpl22 is the target of a late nitrogen checkpoint. Once this checkpoint is satisfied, Rpl22 is activated allowing efficient *IME1* mRNA translation by overriding 5′UTR-mediated inhibition.

In metazoans, L22 is involved in B- and T-cell differentiation and suppressing T-cell transformation [41–43], reviewed in [41]. Based on these reports and results described here, the regulation and function of yeast and vertebrate Rpl22 share both similarities and differences. Neither yeast nor vertebrate Rpl22 are required for bulk translation while both control cell differentiation events. However, unlike vertebrate Rpl22 and Rpl22-like1 that exhibit both overlapping and antagonistic activities, the yeast Rpl22 paralogs have similar functions with Rpl22A being more active. These results are most likely explained by the higher *RPL22A* expression levels compared to *RPL22B* [32]. In mice and zebrafish, Rpl22 antagonizes the expression of Rpl22-like1 [24] while deleting *RPL22A* results in increased *RPL22B* transcription. Finally, vertebrate Rpl22 controls developmental process through translation independent mechanisms. In yeast, although a non-translational role for Rpl22A has been reported that helps target specific mRNAs to the bud [32], we find that Rpl22 mediates meiotic entry by translational control of *IME1*.

**Fig. 7:**
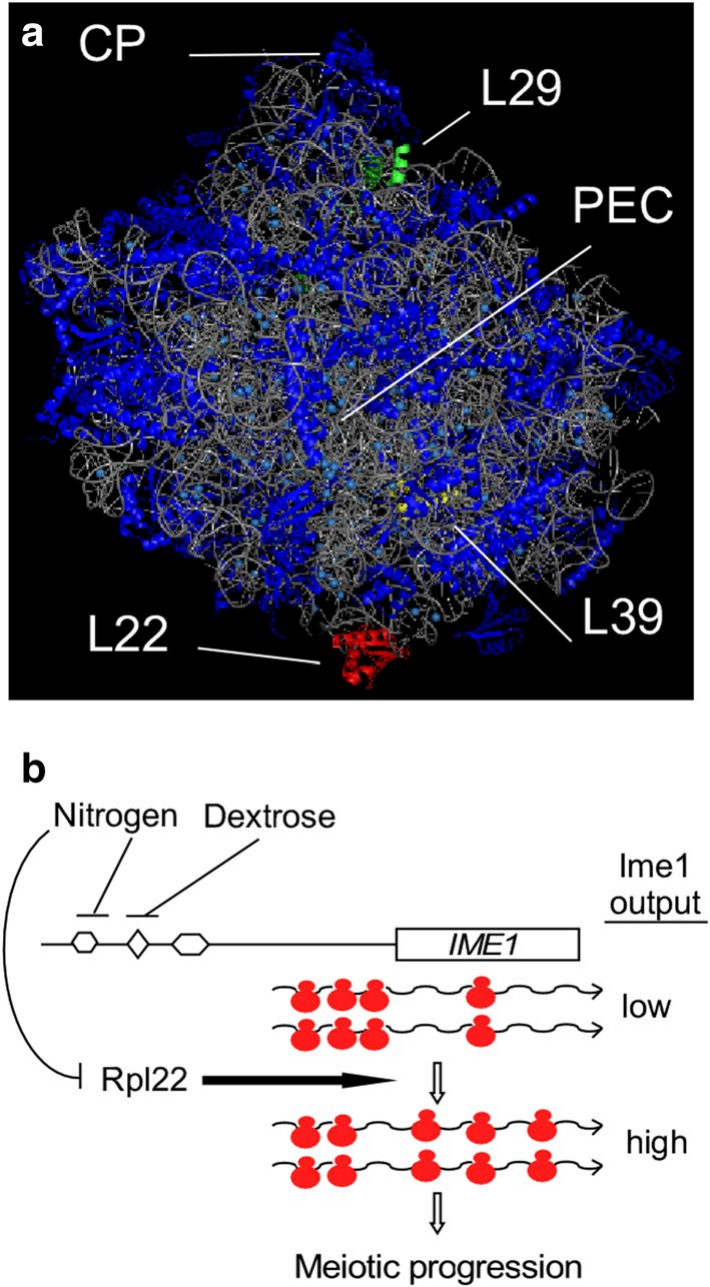
Model for Rpl22 function. **a** Location of Rpl22 (*red*), Rpl29 (*green*), Rpl39 (*yellow*), central protuberance (CP) and peptide exit channel (PEC) are indicated. **b** Cartoon of *IME1* gene structure with upstream promoter elements repressed by dextrose or nitrogen. Ribosome stalling on the 5′UTR is indicated resulting in low Ime1 production. Release of Rpl22 activity from nitrogen repression allows full *IME1* mRNA translation and increased Ime1 production sufficient for meiotic progression. **a** Large subunit structure adapted from [16]

We identified a strong halfmer phenotype in *rpl22∆* mutants growing in rich medium. Mutations in another large subunit ribosomal protein, Rpl29, also demonstrates a “halfmer” phenotype [34]. However, unlike *rpl22∆* mutants, *rpl29∆* strains do not exhibit a reduction in free 60S subunit accumulation. The finding that Rpl29 is located on the 40S interaction face of the large subunit (Fig. 7a) is consistent with a stabilizing role in subunit association. Conversely, we find that Rpl22 is required to maintain the normal 60S:40S ratio suggesting that loss of 60S subunit concentration contributes to this phenotype. This possibility is supported by the finding that the halfmer population and the 60S:40S imbalance is largely missing in *rpl22∆* sporulating cultures. These results suggest that the *IME1* mRNA translation defect and the halfmer phenotype are most likely independent events. How does Rpl22 enhance 60S subunit stability? Rpl22 is located away from 40S interaction and binds the 25S rRNA (Fig. 7a). Therefore, one model is that Rpl22 assists 60S subunit assembly by interacting with the rRNA. Alternatively, its role may be in subunit maintenance by protecting the 25S rRNA from nuclease attack. These two activities are not mutually exclusive leaving open the possibility that the reduction in 60S subunit concentration is the result of multiple factors. Regardless of the mechanism, this phenotype only occurs in rapidly dividing cells that normally maintain high levels of free subunits to accommodate elevated translation rates. This may suggest that Rpl22 is not involved in 60S stability when part of the 80S particle. Understanding how the cell adjusts free subunit content based on high or low metabolism will provide insight into this question.

Gene transcription is regulated by signaling networks responding to both intrinsic and extrinsic stimuli [44]. Similarly, *IME1* transcription is controlled by the PKA and TOR signaling pathways that monitor the nutritional status of the cell [45–48]. *IME1* transcription displays three regulatory states namely off, low and high-level expression [49]. These states indicate cellular conditions of mitotic cell division, conditions permissive to enter meiosis or meiotic induction itself, respectively. We find a similar tiered structure for *IME1* mRNA translation as well. Cells growing in the absence of glucose but still sensing nitrogen fail to translate *IME1* mRNA although the transcript is present (compare 0 h timepoints, Figs. 3, 5). Transfer to sporulation medium induces a low level of *IME1* mRNA translation (3–6 h, Fig. 5). Ime1 levels then elevate (9–12 h) to a threshold sufficient to induce Ume6 destruction and subsequent induction of early meiotic genes such as *IME2*. However, this induction step requires Rpl22 placing specialized translation into the meiotic induction pathway. These results suggest a model that the nitrogen signal inhibits Rpl22 function thus preventing Ime1 accumulation to a level sufficient to induce meiosis (Fig. 7b). Only when the nitrogen signal is completely removed does Rpl22 become fully functional. Consistent with this model, four phosphorylation sites on Rpl22 have been mapped including potential MAPK/Cdk and caseine kinase recognition sites. This step may provide the cell another safeguard to insure that conditions are correct to enter meiosis. It has been previously described that the small subunit and translation initiation factors receive signals that control the initiation process [50–52]. Our results suggest that the large ribosomal subunit is also a recipient of such signals, allowing increased translational efficiency of developmental mRNAs.

Our results indicate that Rpl22 mediates *IME1* mRNA translation through its large 5′UTR. This result is consistent with previous studies that identified this region as important for meiotic translation [10, 11]. The 5′UTR is an important regulatory element in the translation of developmentally regulated loci such as the HOX genes in vertebrates [53, 54]. In the case of HOXa5 and HOXa9, the 5′UTR utilizes an internal ribosome entry site (IRES) through an Rpl38-dependent mechanism. In addition to meiosis, we demonstrate that Rpl22 is required for both invasive and pseudohyphal growth. Two genes required for these processes (*FLO8*, *FLO11*) also possess long 5′UTRs that utilize IRES elements for translation [55]. These observations reveal a possible role for Rpl22 in IRES utilization. This possibility is supported by the finding that Rpl22 enhances IRES mediated translation in the hepatitis C virus 3′UTR [56].

Another mechanism by which the 5′UTR restricts translation is through the presence of short upstream open reading frames (uORFs) [12]. Scanning 40S subunits recognize these uORFs and initiate translation only to terminate the process after a short peptide is generated [15, 57]. The *IME1* 5′UTR does not contain any uORFs with the canonical AUG start codon. However, a previous study identified ribosomal pausing sites within meiotic 5′UTRs that contain proposed non-canonical sites (e.g., CUG, AUU, GUG) [57]. Examination of the *IME1* 5′ UTR revealed several of these sequences suggesting the possibility that *IME1* mRNA translation may be regulated by ribosome pausing. This possibility is supported by the finding that ribosomal pausing is observed at the 5′UTR in *IME1* mRNA early in development but is lost as cells progress through meiosis [57], see Fig. 7b). As would be predicted, ribosome release from the 5′ UTR occurs coincident with Ime1 protein appearance and the Rpl22 execution point. This model is consistent with our results revealing an initial low-level accumulation of Ime1 followed by a rapid elevation in protein concentration that is dependent on Rpl22.

## Conclusion

Rpl22 is a conserved component of the eukaryotic ribosome that carries out specialized functions in many organisms. We find that Rpl22 is required for adopting hyphal growth characteristics and meiotic entry in *S. cerevisiae*. The latter role is due to Rpl22-dependent translation of the meiotic inducer *IME1* mRNA.

## Abbreviations

mRNA: messenger RNA
UTR: untranslated region
uORF: upstream open reading frame
IRES: internal ribosome entry sites
CHX: cycloheximide
qRT-PCR: quantitative reverse transcriptase-polymerase chain reaction
DAPI: 4′,6-diamidino-2-phenylindole
YPA: yeast extract peptone acetate
YPD: yeast extract peptone dextrose
SPM: sporulation medium
AMV-RT: avian myeloblastosis virus-reverse transcriptase
DEPC: diethylpyrocarbonate
EDTA: ethylenediaminetetraacetic acid
SLAD: synthetic, low-ammonia, dextrose
PVDF: polyvinylidene fluoride
RP: ribosomal proteins
