# Erratum to: Rpl22 is required for* IME1* mRNA translation and meiotic induction in *S. cerevisiae*

**DOI:** 10.1186/s13008-017-0031-z

**Published:** 2017-07-18

**Authors:** Stephen K. Kim, Randy Strich

**Affiliations:** Department of Molecular Biology, Rowan University School of Osteopathic Medicine, Two Medical Center Dr., Stratford, NJ 08055 USA

## Erratum to: Cell Div (2016) 11:10 DOI 10.1186/s13008-016-0024-3

Unfortunately, the original version of this article [1] contained an error. The author name “Stephen K. Kim” was misspelt “Stephen J. Kim”.
